# GAN-based bone suppression using a combined loss function

**DOI:** 10.3389/frai.2026.1761336

**Published:** 2026-03-26

**Authors:** Lukáš Jochymek, Markéta Vašinková, Vít Doleží, Petr Gajdoš

**Affiliations:** Faculty of Electrical Engineering and Computer Science, Department of Computer Science, VSB - Technical University of Ostrava, Ostrava, Czech Republic

**Keywords:** autoencoders, bone suppression, chest radiographs, combined loss function, computer-aided diagnosis, generative adversarial network, U-Net, X-ray image analysis

## Abstract

**Introduction:**

Accurate analysis of chest radiographs (X-rays) is essential for diagnosing diseases such as pneumonia and lung cancer, yet bone structures often obscure critical soft tissues and lesions. From an artificial intelligence perspective, bone suppression can be formulated using different modeling paradigms that reflect distinct assumptions about the task.

**Methods:**

In this study, the problem is addressed as a comparative methodological investigation, and three conceptually different approaches are systematically evaluated within a unified experimental framework: denoising-based regression using autoencoders, structured image-to-image transformation using U-Net architectures, and distribution-based generative modeling using adversarial learning. In addition, the impact of different loss configurations and training regimes on reconstruction quality is examined. An enhanced generative adversarial network (GAN) with improved generator and discriminator components and a combined loss function (Wasserstein, L1, perceptual, and Sobel losses) is proposed to improve structural consistency and preserve soft-tissue appearance.

**Results:**

Model performance was assessed using the peak signal-to-noise ratio (PSNR) and the multi-scale structural similarity index measure (MS-SSIM). Among the evaluated approaches, the GAN achieved the best performance, reaching a PSNR of 44.09 dB and an MS-SSIM of 0.9968, and outperformed recently published methods evaluated on the same dataset.

**Discussion:**

These results highlight the importance of both modeling paradigm selection and loss formulation for achieving structurally consistent bone suppression in chest radiographs.

## 1 Introduction

**Figure 1:**
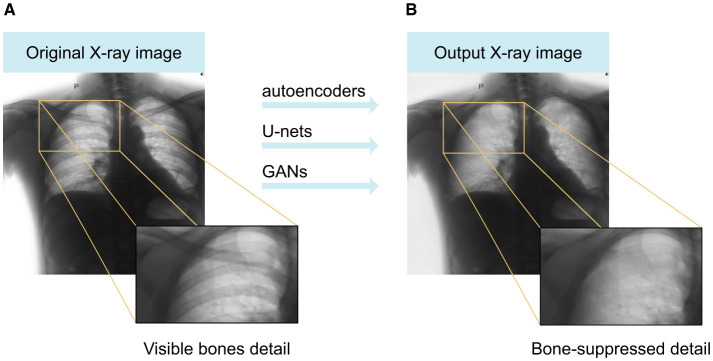
**(A)** Original image from the X-ray, **(B)** output image with removed bones.

Radiographs, commonly referred to as X-ray images, play an important role in medical diagnostics, helping healthcare professionals identify various conditions and guide treatment plans. However, traditional radiographs often present challenges due to the presence of bone structures, which can hinder accurate diagnosis by casting shadows over parts of the lesion, leading to potential misinterpretations. Furthermore, ionizing radiation poses a significant risk to the human body, especially with repeated exposure; therefore, the current trend is to minimize this radiation as much as possible (Hong et al., 2021). However, reducing radiation doses decreases the quality of the resulting images, making diagnosis even more difficult (Uffmann and Schaefer-Prokop, 2009). For this reason, computational image processing techniques for the suppression of bones (ribs) from chest radiographs have become a part of assisted radiology (Schalekamp et al., 2013; Bae et al., 2022) and are an important preprocessing step in computer-aided diagnosis (CAD) (Horváth et al., 2013; Elgohary et al., 2022), contributing to the correct classification of lung disease (Han et al., 2022). This is mainly due to the use of convolutional neural networks, which have become a phenomenon in recent years because they can offer high accuracy and robust performance. We proposed a GAN-based method (Arjovsky et al., 2017; Rani et al., 2022) to generate chest images with bones removed and compared it with other neural network-based methods widely used in image processing, namely autoencoders (Bank et al., 2020; Vincent et al., 2008; Lee et al., 2018) and U-Net (Zhou et al., 2020; Gusarev et al., 2017). To illustrate the potential impact of our method, we provide the following example: Figure 1 presents an original chest radiograph, where bone structures obscure the visibility of lung tissues. The application of our software, as demonstrated in the processed images, effectively removes these bone structures, thereby enhancing the clarity of soft tissue details. This improved visualization may facilitate more precise clinical interpretation of chest radiographs, potentially supporting diagnostic assessment while remaining subject to further clinical validation.

We conducted extensive experiments to evaluate the performance of our bone removal software using various architectures and loss functions. The best results were achieved with the GAN neural network, yielding a Multiscale Structural Similarity Index Measure (MS-SSIM) of **0.9968** and a Peak Signal-to-Noise Ratio (PSNR) of **44.09 db**.

From an artificial intelligence perspective, bone suppression in chest radiographs can be formulated using different modeling paradigms that reflect distinct assumptions about the task. In this study, we address bone suppression as a comparative AI methodology problem and systematically evaluate three conceptually different approaches: denoising-based regression using autoencoders, structured image-to-image transformation using U-Net architectures, and distribution-based generative modeling using adversarial learning. The aim is to provide a unified comparison of these paradigms under a consistent experimental and evaluation framework in order to identify the most suitable modeling strategy for bone suppression. As the differences between methods are often subtle and primarily structural, they are more reliably captured by quantitative metrics than by direct visual inspection at standard resolution. Clinical validation and reader-based studies are therefore considered subsequent research steps that follow the establishment of a robust methodological foundation.

### 1.1 Bones removal dataset

**Figure 2:**
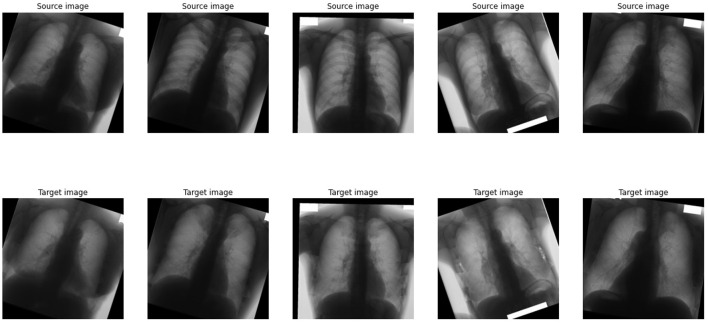
Example of images from augmented JSRT dataset.

In 1998, the Academic Committee of the Japanese Society of Radiological Technology (JSRT) launched an initiative to establish the Standard Digital Image Database, a collection of chest radiographs containing both normal cases and cases with lung nodules. This effort, undertaken in collaboration with the Japan Radiological Society, was made possible through contributions of clinical cases from medical institutions in both Japan and the United States. Over the years, the JSRT database has become a widely utilized resource in medical imaging research, facilitating advancements in image processing, compression techniques, display assessments, Picture Archiving and Communication Systems (PACS), and the development of machine learning-based computer-aided diagnosis (CAD) systems. The database comprises 247 digitized chest radiographs (CXRs), including 154 cases with lung nodules—100 classified as malignant, 54 as benign, and 93 cases without nodules. These images were digitized using a laser scanner at a resolution of 2, 048 × 2, 048 pixels (0.175 mm per pixel) with a 12-bit grayscale depth. The images are stored in big-endian raw format without headers (Shiraishi et al., 2000). To expand the dataset, various augmentation techniques were employed, including affine transformations such as rotation (-10 to 10 degrees), horizontal and vertical translation (-5 to 5 pixels), horizontal flipping, and zooming. Additionally, image processing techniques such as median filtering, maximum and minimum filtering, and unsharp masking were applied. As a result, the dataset was expanded to 4081 image pairs at a resolution of 1, 024 × 1, 024 pixels, consisting of both the original CXRs and their corresponding bone-suppressed versions (Rajaraman et al., 2021). While this augmentation strategy increases training diversity, it does not substitute for population-level variability or independent institutional datasets. An example of these images is provided in Figure 2, where the upper image displays a chest radiograph containing lung nodules, and the lower image presents the same radiograph with suppressed bone structures.

## 2 Methods

In this section, we present three deep learning strategies for bone suppression, each of which has been custom-implemented to align with the architectures proposed in their foundational works. First, we designed a custom autoencoder architecture inspired by the original model (Gondara, 2016), modifying certain layers and hyperparameters to better suit our objective of bone suppression. Second, we developed a U-Net (Ronneberger et al., 2015) that closely follows the encoder-decoder structure described by Ronneberger et al. but includes adjustments for improved handling of subtle bone features. Lastly, we constructed a custom GAN model by combining key ideas from both the SFRM-GAN (Rani et al., 2022) and Wasserstein GAN (Arjovsky et al., 2017) frameworks, ensuring that it could learn to suppress bones robustly without sacrificing overall image fidelity.

### 2.1 Autoencoder

An autoencoder is a specific type of neural network that is primarily designed to encode input data into a compressed representation and then reconstruct it to resemble the original as closely as possible. Autoencoders are widely used in applications such as dimensionality reduction (Meng et al., 2017; Wang et al., 2014), classification (Luo et al., 2017), anomaly detection (Sakurada and Yairi, 2014), and noise reduction (Chiang et al., 2019; Bengio et al., 2013; Creswell and Bharath, 2019). For noise reduction, denoising autoencoders are frequently employed. In these models, a noise layer is introduced immediately after the input layer, followed by training the hidden and reconstruction layers with data that include noise. A denoising autoencoder is an extension of a conventional autoencoder, in which the model learns to map corrupted data to uncorrupted data by minimizing the loss function between their representations (Bank et al., 2020). The learning objective of this model is to recover the clean input from the noisy and corrupted version. The corrupted signal, $\tilde{X}$, is generated from the clean signal, *X*, by random mapping $\tilde{X}\sim q_{D}\left(\tilde{X}|X\right)$ (Li et al., 2023).

In this work, a convolutional denoising autoencoder (CDAE) (Gondara, 2016) was used to address the challenge posed by the ribs, which act as unwanted noise in chest radiographs. Unlike traditional fully connected layers, the convolutional autoencoder employs convolution and pooling operations within the convolutional neural network, effectively preserving two-dimensional spatial information (Li et al., 2023). It is well suited for images, as it preserves spatial locality by sharing weights across all input locations. Gondara et al. have shown Gondara (2016) that CDA can be used for efficient denoising of medical images by using small training datasets to achieve good performance. The ability of CDAEs to effectively denoise medical images using a model trained on a relatively small training dataset has also been demonstrated by Lee et al. (2018) on chest radiograph images. Kalisz and Marczyk (2021) developed a CDAE-based architecture and trained a model on the Bone Suppression dataset to remove bones from chest radiographs (COVIDx dataset). Ahmed et al. (2021) employed a stacked convolutional autoencoder (SCAE), an improved form of a basic denoising autoencoder with integrated convolutional layers, to reduce noise in two-dimensional gel electrophoresis (2-DGE) images.

The input to the autoencoder consists of a 1, 024 × 1, 024 × 1 image normalized to the range [0, 1].

**Figure 3:**
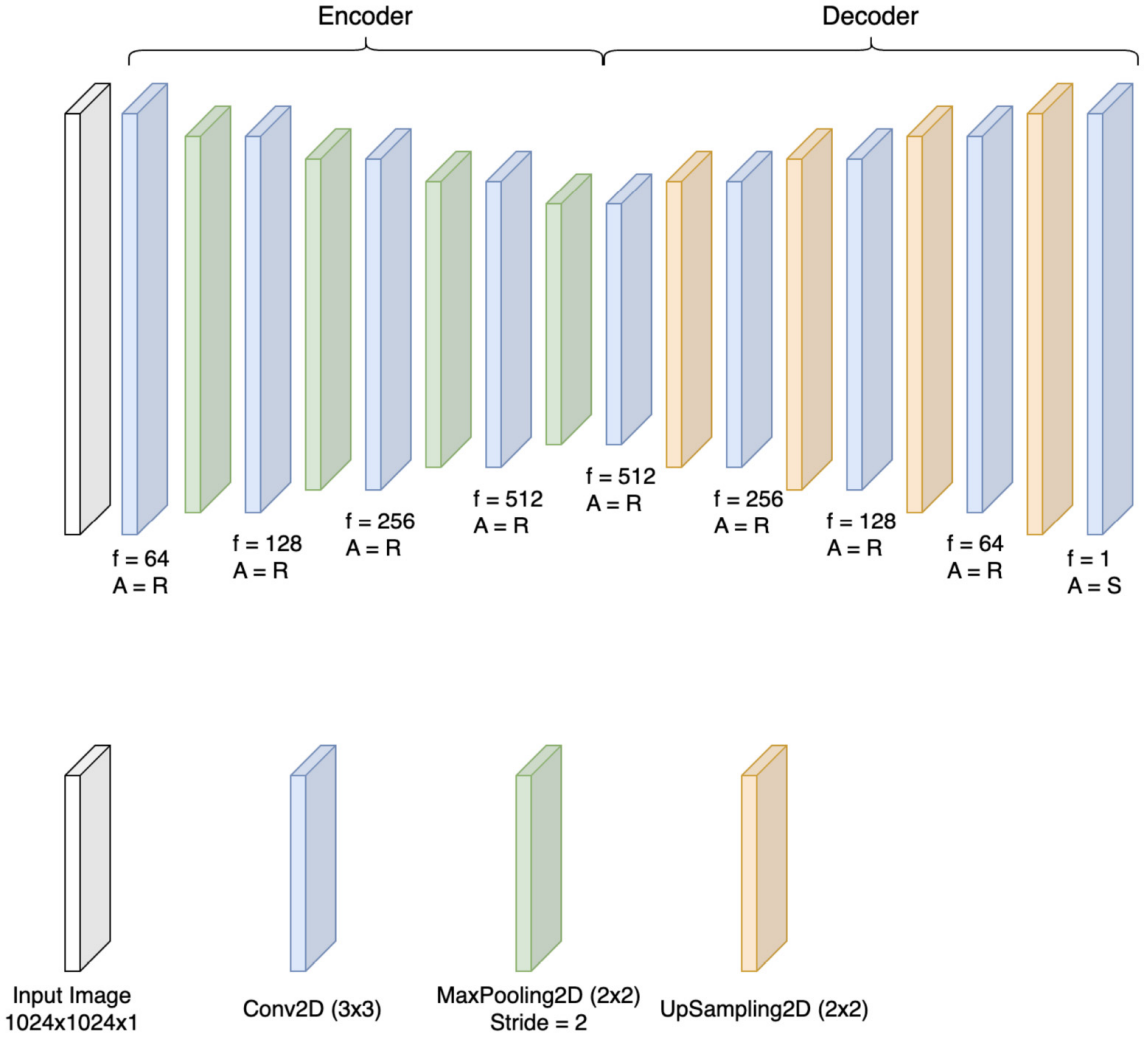
Convolutional autoencoder architecture for bone suppression: the encoder-decoder framework begins with an encoder that uses convolutional (Conv2D) and pooling (MaxPooling2D) layers to extract and compress image features, focusing on distinguishing bones. Each layer progressively captures more abstract features. The decoder then reconstructs the image using upsampling layers (UpSampling2D), enhancing soft tissue visibility and reducing bone prominence. Here, f = number of filters and A = activation function either ReLU or Sigmoid.

The convolutional autoencoder architecture in Figure 3 used for bone suppression operates on an encoder-decoder framework. The encoder, composed of convolutional and cluster layers, extracts key features while compressing the image into a compact representation, focusing on differentiating bones from other structures. As the image moves through these layers, progressively abstract features are identified, from basic edges to more complex structures like bone tissue.

The decoder then reconstructs the image in order to enhance the visibility of soft tissue while reducing bone prominence. By focusing on features related to soft tissues, the autoencoder effectively separates bone characteristics, producing a clearer image for diagnostic evaluation.

### 2.2 U-Net

**Figure 4:**
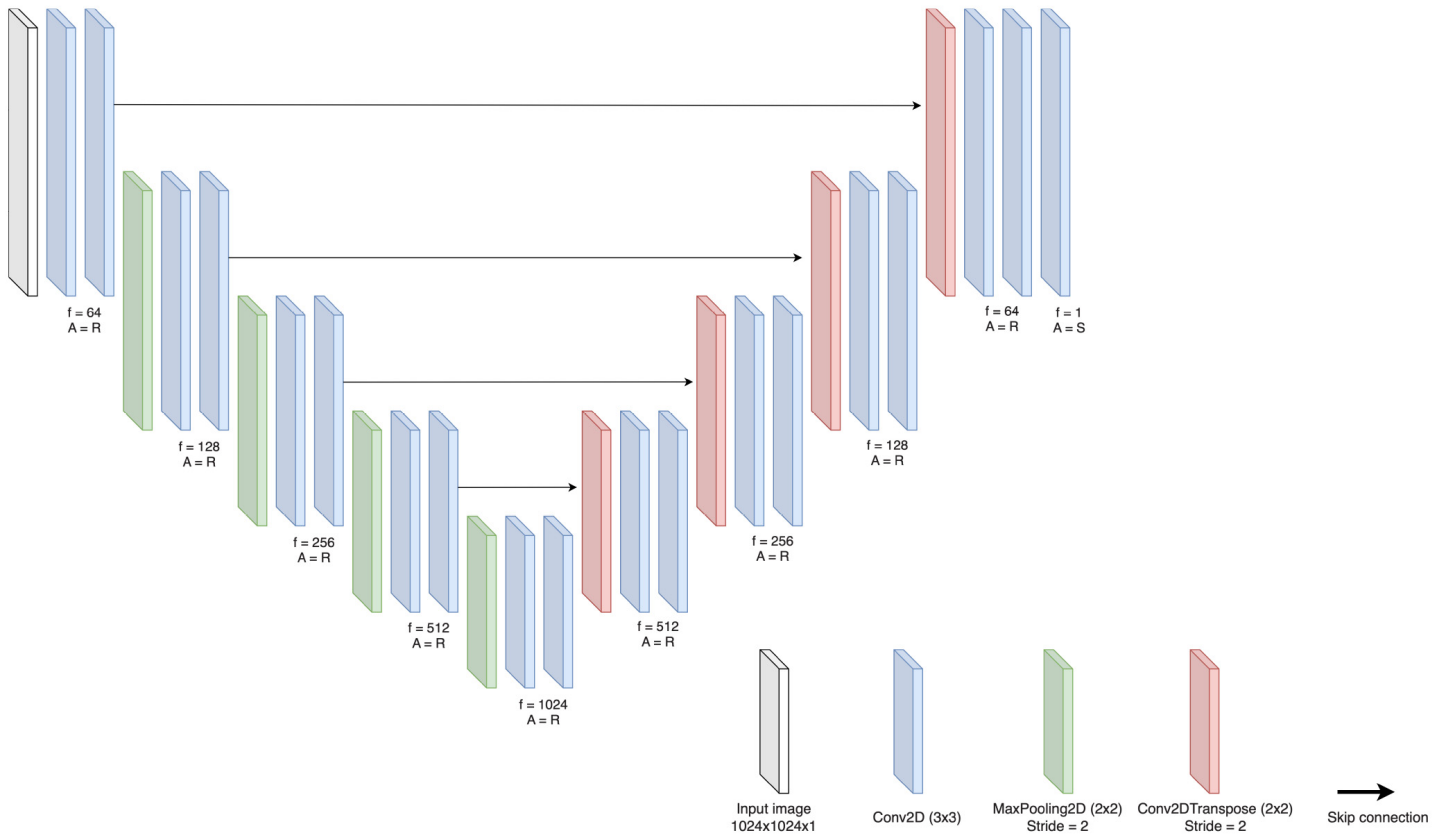
The proposed U-Net architecture uses an encoder-decoder structure with skip connections. The encoder uses convolutional and pooling layers to capture progressively abstract image features, essential to distinguish bones from soft tissue. At the bottleneck, the image is compressed into a compact and detailed representation. The decoder then reconstructs the image with minimized bone features, using skip connections to retain fine-grained details. f = number of filters and A = activation function ReLU or Sigmoid.

U-Net is an encoder-decoder model that is widely used in segmentation tasks, including the segmentation of chest radiographs (Zheng et al., 2021; Osadebey et al., 2021; Novikov et al., 2018). Wang et al. (2020) proposed the multitask dense connection U-Net (MDU-Net) to address the challenge of bone segmentation from a chest radiograph. Their method combines the U-Net multiscale feature fusion approach, the DenseNet dense connection, and the multitasking mechanism to construct the proposed MDU-Net. For bone removal, Cardenas et al. (2021) used a U-Net architecture, demonstrating that bone removal from chest radiographs is feasible using a CNN combined with a patch-based approach by effectively segmenting and excluding bone structures. To succeed in the bone suppression task, we must input chest radiographs in which bone structures are prominent and must be distinguished from other tissues. The U-Net architecture can be effective in this context, with its unique up-sampling and down-sampling layers. In this case, we are using an original model from the paper U-Net: Convolutional Networks for Biomedical Image Segmentation (Ronneberger et al., 2015) together with various backbones of the Segmentation Model library (Iakubovskii, 2019). The input image to the U-Net is 1, 024 × 1, 024 × 1 normalized in the range of [0, 1]. The proposed architectures can be seen in Figure 4.

At each layer of the encoder, the network applies filters to progressively extract image features, beginning with basic edge detection in the initial layers and advancing to more intricate patterns in the deeper layers. This hierarchical feature extraction is essential for distinguishing subtle differences between bones and surrounding tissues in radiographic images.

As the image passes through the encoder, its spatial resolution decreases while the extracted features become more abstract and informative. This trade-off allows the network to focus on the most relevant characteristics, particularly those necessary for identifying and suppressing bone structures. Upon reaching the bottleneck layer—the transitional point between the encoder and decoder—the image is transformed into a compact yet information-rich representation, emphasizing the critical features required for effective bone suppression.

The decoder then reconstructs the image using this refined information, ensuring that bone features are minimized while preserving other anatomical structures. The encoder's precise extraction of bone-related features plays a crucial role in this process, enabling the decoder to accurately suppress bones in the final output. This systematic approach allows the network to accurately identify and modify bone structures while maintaining the visibility of other essential anatomical details. The synergy between the encoder and decoder in this process makes U-Net particularly effective for bone suppression in radiographic imaging, demonstrating a sophisticated approach that goes beyond conventional segmentation to achieve targeted image transformation.

### 2.3 Feature pyramid networks

In addition to the original U-Net architecture, we evaluated Feature Pyramid Networks (FPN) (Lin et al., 2017) within the same segmentation framework. FPN augments a backbone CNN with a top-down pathway and lateral 1 × 1 convolutional connections to enable multi-scale feature aggregation. Instead of constructing explicit image pyramids, FPN reuses intrinsic hierarchical representations of the backbone (e.g., ResNet or EfficientNet), providing semantically strong feature maps at multiple resolutions with moderate computational overhead.

In contrast to U-Net (Ronneberger et al., 2015), which employs a symmetric encoder-decoder architecture with full-resolution decoding and dense skip connections for pixel-level reconstruction, FPN performs additive multi-scale fusion on backbone feature maps and applies task-specific prediction layers to aggregated representations. Within the Segmentation Models library (Iakubovskii, 2019), FPN is adapted for dense prediction; however, compared to U-Net, it emphasizes hierarchical semantic aggregation rather than explicit spatial reconstruction, leading to different trade-offs between localization precision and computational complexity.

### 2.4 Generative adversarial networks

**Figure 5:**
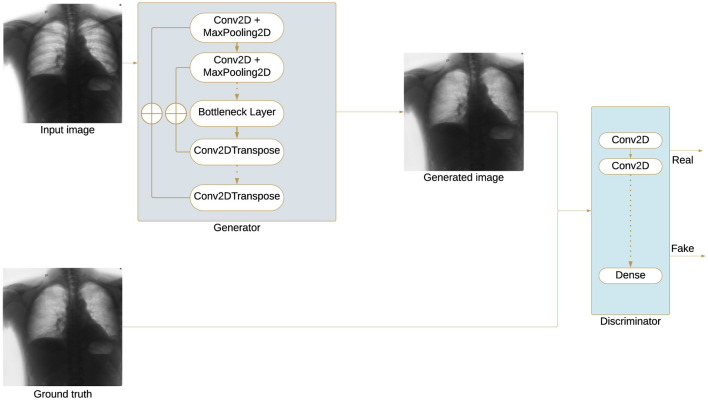
Bone suppression GAN architecture: The generator processes the input X-ray through convolutional and pooling layers to extract features, compresses them in a bottleneck layer, and reconstructs a bone-suppressed image via upsampling. The discriminator classifies the generated and real images, distinguishing between real and synthetic outputs.

Generative adversarial networks (GANs) consist of two components: the generator (G) and the discriminator (D). Given a specific condition or input, the role of the generator is to produce realistic images that ideally should be indistinguishable from the target images (Creswell et al., 2018). Conversely, the discriminator is responsible for distinguishing between the generated images and the actual target images. In the context of bone suppression, the generator is expected to learn how to generate images in which bones are effectively suppressed (Figure 5).

Rani et al. (2022) addressed the bone suppression task by modifying the architectures of both the generator and discriminator in the Pix2Pix model. Their approach improved performance and training stability by integrating the discriminator into the Wasserstein GAN framework, further enhanced with a Gradient Penalty. Similarly, Han et al. (2022) introduced a GAN-based disentanglement learning framework, termed Rib Suppression GAN (RSGAN), to perform rib suppression by leveraging anatomical knowledge embedded in unpaired CT images. Their method focuses on predicting the residual map between the input chest X-ray (CXR) and its corresponding rib-suppressed version.

Our proposed architecture is inspired by the Wasserstein GAN (Arjovsky et al., 2017) and the Spatial Feature and Resolution Maximization GAN (SFRM-GAN) (Rani et al., 2022). Due to the incorporation of various loss functions and additional computational constraints, the input images are resized to 512 × 512 × 3 and normalized to the range of [−1, 1].

#### 2.4.1 Discriminator

**Figure 6:**
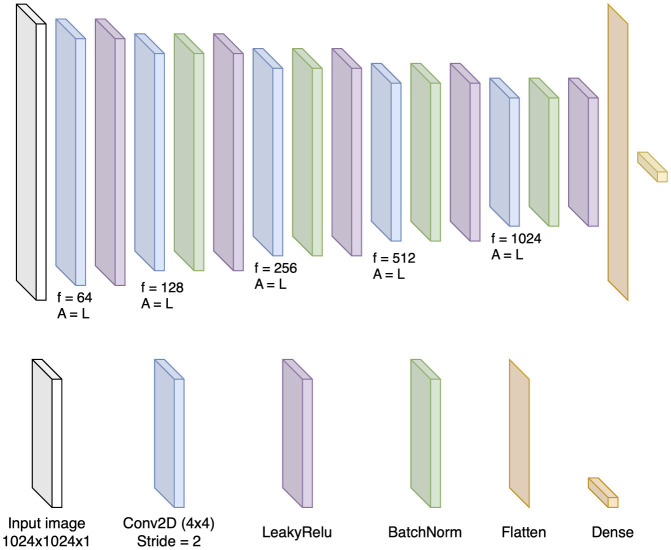
Discriminator architecture with down-sampling Conv2D layers, batch normalization, and a linear activation function. Filters and strides are noted for each layer. The f = a number of filters and A = activation function, which is, in this case, linear.

The architecture of the discriminator, illustrated in Figure 6, progressively downsamples the input to determine whether an image is real or generated (real vs. fake); in our case, whether the image is suppressed or synthesized. To enhance training stability and accelerate convergence, batch normalization is applied after every convolutional layer except the first one. Each convolutional layer employs a stride of 2, effectively halving the spatial dimensions of the feature maps at each stage. This systematic reduction in size enables efficient downsampling of the input image while preserving essential features for discrimination.

#### 2.4.2 Generator

**Figure 7:**
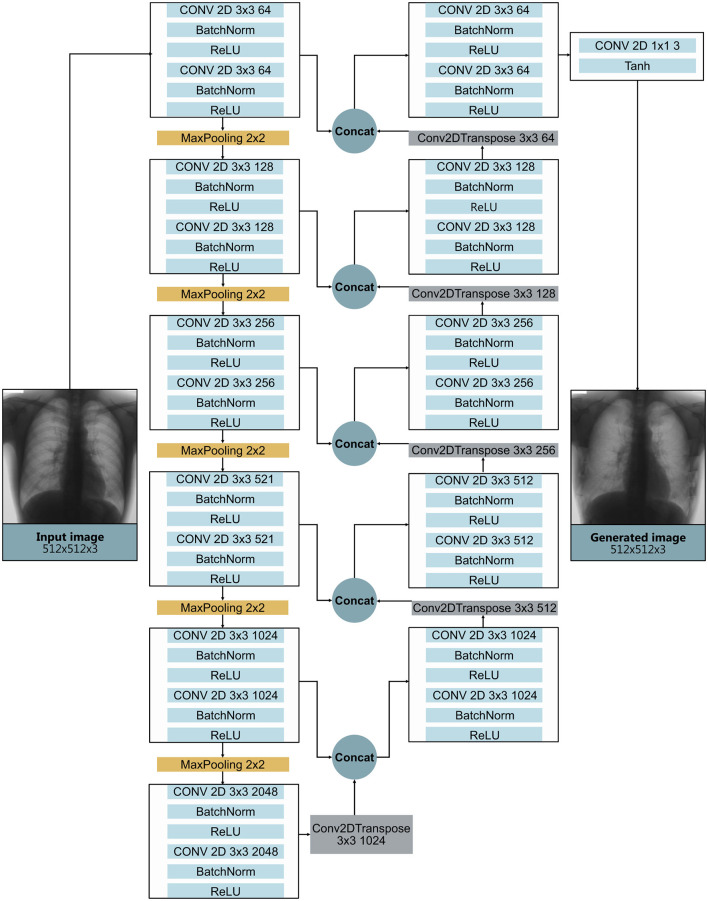
Generator architecture with downsampling path (**left**) and an upsampling path (**right**) with skip connections between corresponding layers. Convolutional blocks progressively extract features, culminating in a bottleneck layer. Transpose convolutions in the upsampling path restore spatial resolution, while skip connections preserve fine details, enabling precise feature localization.

The generator architecture in Figure 7 is modeled primarily after the U-Net framework, which has demonstrated strong performance in bone suppression tasks, as evidenced by the results presented in Section 3.2. The architecture employs a series of convolutional blocks to progressively extract and encode complex features at multiple hierarchical levels, culminating in the bottleneck layer. Following this, transpose convolutions are applied to incrementally double the spatial dimensions of the feature maps. To maintain spatial consistency and preserve critical features that may be lost during downsampling, the upsampled outputs are concatenated with corresponding feature maps from the encoder path through skip connections.

During the GAN training process, the generator and discriminator networks are trained simultaneously. Automatic differentiation, implemented using gradient tape, plays a key role in updating the network weights efficiently. The generator produces sample images, while the discriminator evaluates them, distinguishing between real and generated images. Both networks continuously adjust their weights based on the computed gradients, improving their performance through an iterative feedback loop. This ongoing refinement continues until the generator successfully produces images with effectively suppressed bone structures.

### 2.5 Loss functions and evaluation metrics

We need some metrics to assess how well a model has performed; in our case, we have selected two evaluation metrics: the Structural Similarity Index Measure and the Peak Signal-to-Noise Ratio. Loss functions are either a combination of the evaluation metrics used as losses or completely different functions. These metrics provide quantitative ways to assess the effectiveness of bone suppression algorithms and are also used in other publications (Lam et al., 2022; Kalisz and Marczyk, 2021; Gusarev et al., 2017; Ren et al., 2021; Rani et al., 2022).

#### 2.5.1 Evaluation metrics

It is essential to define suitable evaluation metrics to measure and compare the performance of the models. In this study, we will employ the same metrics used in previous research, namely the Multi-Scale Structural Similarity Index Measure (MS-SSIM) and the Peak Signal-to-Noise Ratio (PSNR).

#### 2.5.2 Multi-scale structural similarity index measure (MS-SSIM)

The generated images are compared with the target images in terms of their structural similarity. A high MS-SSIM value indicates a more remarkable similarity in the structure of the target and the generated images (Hore and Ziou, 2010). MS-SSIM is a crucial metric for assessing image quality, extending beyond the traditional Structural Similarity Index Measure by incorporating multiple scales and structural features. It evaluates how well structural information is preserved between a reference image and a distorted version, offering a more comprehensive assessment of perceptual image quality. The Structural Similarity Index Measure equation consists of three components: i) luminance comparison function, defined as $l\left(x,y\right)=\frac{\left(2\mu_{x}\mu_{y}+c_{1}\right)}{\left(\mu_{x}^{2}+\mu_{y}^{2}+c_{1}\right)}$, ii) contrast comparison function, defined as $c\left(x,y\right)=\frac{2\sigma_{x}\sigma_{y}+c_{2}}{\sigma_{x}^{2}+\sigma_{y}^{2}+c_{2}}$, and iii) structure comparison function, defined as $s\left(x,y\right)=\frac{\sigma_{xy}+c_{3}}{\sigma_{x}\sigma_{y}+c_{3}}$, where *x* is the reference image (ground truth), *y* is the distorted image (predicted), μ_*x*_, μ_*y*_ are the pixel sample means of *x* and *y*, $\sigma_{x}^{2},\sigma_{y}^{2}$ are the variances of *x* and *y*, and σ_*xy*_ is the covariance of *x* and *y*; *c*_1_, *c*_2_, *c*_3_ are the variables to stabilize the division with a weak denominator. The SSIM Equation 1 is given as a weighted combination of these comparative measures, formulated as:


$$\begin{array}{l} \text{SSIM}\left(x,y\right)=l{\left(x,y\right)}^{\alpha }\cdot c{\left(x,y\right)}^{\beta }\cdot s{\left(x,y\right)}^{\gamma } \end{array}$$ (1)

where α, β, and γ are the weights of the appropriate comparison functions. By setting these weights to 1 (Equation 2), the formula simplifies to:


$$\begin{array}{l} \text{SSIM}\left(x,y\right)=\frac{\left(2\mu_{x}\mu_{y}+c_{1}\right)\left(2\sigma_{xy}+c_{2}\right)}{\left(\mu_{x}^{2}+\mu_{y}^{2}+c_{1}\right)\left(\sigma_{x}^{2}+\sigma_{y}^{2}+c_{2}\right)}. \end{array}$$ (2)

After adding multiple scales and structural characteristics (Equation 3), the MS-SSIM is expressed as follows:


$$\begin{array}{l} \text{MS-SSIM}\left(x,y\right)={\left[l_{M}\left(x,y\right)\right]}^{\alpha }\cdot \overset{M}{\underset{j=1}{\prod }}{\left[c_{j}\left(x,y\right)\right]}^{\beta }\cdot {\left[s_{j}\left(x,y\right)\right]}^{\gamma } \end{array}$$ (3)

where *l*_*M*_(*x, y*) is the luminance comparison function on the M-th scale (coarsest), *c*_*j*_(*x, y*) is the contrast comparison function on the j-th scale, *s*_*j*_(*x, y*) is the structure comparison function on the j-th scale, and M is the number of scales used for the calculation of MS-SSIM.

#### 2.5.3 Peak signal-to-noise ratio (PSNR)

The PSNR quantifies the relationship between the highest possible signal strength, represented by the original image, and the noise level, which arises from differences between the original and processed images (Equation 4). This value is measured in decibels (dB). A higher PSNR indicates greater similarity between the original and processed images, signifying better image quality. In contrast, a lower PSNR suggests increased distortion or noise in the reconstructed image (Hore and Ziou, 2010).

The PSNR is expressed as follows:


$$\begin{array}{l} \text{PSNR}=10\times \log_{10}\frac{{\text{MAX}}^{2}}{\text{MSE}} \end{array}$$ (4)

where MAX is the maximum possible pixel value of the image, and MSE is the result of the Mean Squared Error (MSE), a common metric for measuring the difference between actual and predicted values. It is calculated as $MSE=\frac{1}{n}\overset{n}{\underset{i=1}{\sum }}{\left(y_{i}-p_{i}\right)}^{2}$, where *n* is the number of observations in the dataset, *y*_*i*_ is the ground truth value, and *p*_*i*_ is the predicted value.

PSNR and MS-SSIM are used as the primary evaluation metrics, as they better reflect perceptual and structural image quality in bone suppression tasks. MSE is additionally reported in selected tables to enable direct comparison with previously published studies, where this metric is commonly provided, while MAE is not included due to its strong correlation with MSE and limited additional interpretative value in this reconstruction setting.

#### 2.5.4 Loss functions

The Mean Squared Error (MSE), the Multi-Scale Structural Similarity Index Measure (MS-SSIM), and combinations of pixel-wise losses (MSE or MAE) with MS-SSIM are utilized as loss functions in selected models. The MS-SSIM (Equation 3) metric is defined in section 2.5, and the corresponding loss formulation is denoted as *L*_*MSSSIM*_.

Pixel-wise losses quantify intensity differences between predicted and reference images. The Mean Squared Error (MSE) (Equation 5), equivalent to the *L*_2_ loss, penalizes larger deviations more strongly and is defined as


$$\begin{array}{l} MSE=\frac{1}{n}\overset{n}{\underset{i=1}{\sum }}{\left(y_{i}-p_{i}\right)}^{2}, \end{array}$$ (5)

where *n* is the number of observations, *y*_*i*_ denotes the ground truth value, and *p*_*i*_ represents the predicted value. In contrast, the Mean Absolute Error (MAE) (Equation 6), corresponding to the *L*_1_ loss, computes the mean absolute deviation,


$$\begin{array}{l} MAE=\frac{1}{n}\overset{n}{\underset{i=1}{\sum }}|y_{i}-p_{i}|, \end{array}$$ (6)

and is less sensitive to outliers, which can help preserve fine structural details in lung regions.

To jointly enforce pixel-level fidelity and structural similarity, *L*_1_ or *L*_2_ is combined with *L*_*MSSSIM*_ in a Mixed Loss formulation introduced by Gusarev et al. (2017). The resulting loss functions are defined as


$$\begin{array}{l} L_{\text{Mix}L_{1}}=\alpha \cdot L_{MSSSIM}+\left(1-\alpha \right)\cdot L_{1}, \end{array}$$ (7)

$$\begin{array}{l} L_{\text{Mix}L_{2}}=\alpha \cdot L_{MSSSIM}+\left(1-\alpha \right)\cdot L_{2}, \end{array}$$ (8)

where α is a weighting hyperparameter controlling the balance between structural similarity and pixel-wise accuracy (Equations 7, 8).

Each component of the composite loss addresses a different requirement of the bone suppression problem. Pixel-wise losses (*L*_1_ or *L*_2_) enforce intensity-level similarity between generated and reference images: *L*_1_ tends to better preserve fine soft-tissue textures, while *L*_2_ promotes smoother global intensity transitions that may reduce residual rib artifacts. The MS-SSIM term supports the preservation of multiscale structural information in lung regions.

Within the GAN framework, additional components are used. The Perceptual Loss encourages similarity in a high-level feature space to maintain a realistic soft-tissue appearance, while the Sobel Loss enforces edge consistency, helping to attenuate high-frequency bone edges while preserving diagnostically relevant anatomical boundaries. A full ablation analysis of all loss components would require training multiple GAN variants and is therefore beyond the scope of this study; such an analysis is considered future work.

Although *L*_2_ is convex and differentiable, making it suitable for stable optimization, it may lead to overly smooth reconstructions. Therefore, *L*_1_ is also evaluated, as it has been reported to better preserve contrast and luminance details, often producing sharper outputs than *L*_2_ (Rani et al., 2022). Since the relative effectiveness of *L*_1_ and *L*_2_ for bone suppression has not been conclusively established, both variants are experimentally investigated in this study.

#### 2.5.5 Loss functions for the generator and the discriminator

The concept of Wasserstein Loss (Arjovsky et al., 2017) is derived from the Wasserstein distance, also known as the Earth-Mover's distance. It is calculated by taking the average of the critic's outputs for real data and subtracting the average of its outputs for generated data. Here, the term “critic” refers to the discriminator in the Wasserstein GAN framework, which estimates a continuous score rather than a binary classification probability. The critic aims to maximize this difference by assigning higher scores to real data and lower scores to generated data, whereas the generator strives to minimize this difference by producing data that the critic perceives as real. The Equation 9 used to compute the Wasserstein Loss is as follows:


$$\begin{array}{l} L_{\text{cGAN}}\left(G,D\right)=\frac{1}{n}\overset{n}{\underset{i=1}{\sum }}\left[D\left(X_{i},Y_{i}^{\prime }\right)-D\left(X_{i},Y_{i}\right)\right] \end{array}$$ (9)

where *L*_cGAN_(*G, D*) is the loss of the critic, *G* is the generator, and *D* is the critic; $\frac{1}{n}\overset{n}{\underset{i=1}{\sum }}$ is an average for all *n* samples in the batch; $D\left(X_{i},Y_{i}^{\prime }\right)$ is the output of the critic for the real data pair, and *D*(*X*_*i*_, *Y*_*i*_) is the output of the critic for the generated data pair.

This loss function is consistently applied to the discriminator across all experiments and is also incorporated into the generator's loss formulation. Four distinct loss functions derived from the Wasserstein distance are evaluated. As the complexity of the loss function increases, a larger volume of training data is required, as discussed in Section 3.3.

For the generator, the following loss functions are used and tested:

Wasserstein + L1/L2 loss


$$\begin{array}{l} L_{\text{cGAN}}\left(G\right)=\frac{1}{n}\overset{n}{\underset{i=1}{\sum }}D\left(X_{i},Y_{i}^{\prime }\right)+\delta \left[\alpha L_{L1/L2}\right] \end{array}$$ (10)

Wasserstein + L1/L2 loss + Perceptual Loss


$$\begin{array}{l} L_{\text{cGAN}}\left(G\right)=\frac{1}{n}\overset{n}{\underset{i=1}{\sum }}D\left(X_{i},Y_{i}^{\prime }\right)+\delta \left[\alpha L_{L1/L2}+\beta L_{\text{Perceptual}}\right] \end{array}$$ (11)

Wasserstein + L1/L2 loss + Perceptual Loss + Sobel Loss


$$\begin{array}{l} L_{\text{cGAN}}\left(G\right)=\frac{1}{n}\overset{n}{\underset{i=1}{\sum }}D\left(X_{i},Y_{i}^{\prime }\right)+\delta \left[\alpha L_{L1/L2}+\beta L_{\text{Perceptual}}+\gamma L_{\text{Sobel}}\right] \end{array}$$ (12)

where $\frac{1}{n}\overset{n}{\underset{i=1}{\sum }}D\left(X_{i},Y_{i}^{\prime }\right)$ is the average score that the discriminator gives to the generated data across *n* samples, *X*_*i*_ is the conditioning input, and $Y_{i}^{\prime }$ is the generator-generated output; δ, α, β, γ are the weight parameters for the additional loss components; α*L*_*L*1/*L*2_ is the result of the *L*_1_ or *L*_2_ multiplied by the weight parameter α. The loss formulations in Equations 10–12 can be interpreted as specific instances of a unified composite objective, where simpler variants are obtained by appropriately setting the weighting coefficients (e.g., β = 0 and/or γ = 0). *L*_Perceptual_ is the loss function, also known as VGG-19 Loss, which employs the VGG-19 model pre-trained on ImageNet. This function enhances image synthesis quality by ensuring that the generated feature maps closely resemble those of the target image. It achieves this by measuring the Euclidean distance between the feature representations of the generated and target images, thereby improving information retention and reducing artifacts. The model can also be used with other deep learning architectures, such as ResNet50 and ResNet101, provided they are trained within the appropriate image domain (Johnson et al., 2016). The result of *L*_Perceptual_ is multiplied by the weight parameter β. *L*_Sobel_ is a loss function inspired by edge detection techniques. It calculates errors by comparing the gradient maps of the generated and target images in both the *X* and *Y* directions, effectively capturing discrepancies in image gradients (Lu and Chen, 2022). The result of *L*_Sobel_ is multiplied by the weight parameter γ. For all experiments, including all combinations of loss functions, the hyperparameters are defined as δ = 10, 000, α = 1, β = 10, and γ = 10. The primary objective of the loss function, which integrates Wasserstein Loss, *L*_1_/*L*_2_ loss, Perceptual Loss, and Sobel Loss, is to capture both low- and high-frequency details within the image. This approach is intended to enhance detail preservation, ensuring that essential structures remain intact even after bone suppression.

## 3 Results

For each training dataset size, multiple loss formulations were evaluated, and only the best-performing configuration, selected according to MS-SSIM and PSNR, is reported in Tables 1 and 5. The reported training times should therefore be interpreted as approximate indicators of relative computational demand rather than exact benchmarks.

**Table 1:** Best results for autoencoder experiments.

**Train/test size**	**Loss function**	**Training time (s)**	**MS-SSIM**	**PSNR**
400/100	*L*_Mix_*L*__2__	1 612	0.9805	31.332
1,000/100	*L*_*MSSSIM*_	3 853	0.9827	31.935
**3,980/100**	***L***_Mix_*L*__1__	**27 606**	**0.9847**	**32.850**

The best results are highlighted in bold.

The implementation was carried out using Python 3.6.8, TensorFlow 2.6.2, Segmentation Models v.1.0.1, and CUDA 11.4. The hardware setup included 20 × Intel(R) Xeon(R) Gold 6154 CPUs (@ 3.00 GHz), 2 × NVIDIA TESLA V100 GPUs (32 GB), and 7.3 TB of RAM. The system operated on RedHat 6.0.11.

### 3.1 Results for autoencoders

Since there are many experiments, showing only the best result according to the dataset size should help improve readability. Training of all models uses the Adam optimizer (Kingma and Ba, 2017), configured with a fixed learning rate of 0.001. Furthermore, a decay factor of 0.5 is employed with a patience parameter set to 5 in relation to the validation loss.

As shown in Table 1, the best-performing model was trained on 3,980 samples using the Mixed Loss with the *L*_1_ loss function (*L*_Mix_*L*__1__), with a training duration of approximately 7.7 h. The other models, which utilized the Mixed Loss with the *L*_2_ loss function and the MS-SSIM loss function, demonstrated slightly lower performance, suggesting that the number of training samples has only a minor impact on overall effectiveness.

To further enhance performance, the feature extractor from a more complex pre-trained model was integrated, replacing the encoder. Specifically, ResNet50 and EfficientNetB0 were used as backbone architectures. All experiments in this setup were trained with the Mixed Loss using *L*_1_ (*L*_Mix_*L*__1__) since the best results without the pre-trained encoder were achieved with that loss function. Dataset comprised of 3,980 training samples and 100 test samples, as summarized in Table 2.

**Table 2:** Results for autoencoder experiments with pre-trained encoder.

**Backbone**	**Training time (s)**	**MS-SSIM**	**PSNR**
ResNet50	20 989	0.9856	31.981
**EfficientNetB0**	**38,989**	**0.9909**	**33.541**

The best results are highlighted in bold.

The findings of this experiment indicate that the use of a pre-trained encoder does not inherently lead to a notable performance gain. While the usage of EfficientNetB0 backbone achieves better performance compared to the previous autoencoder (AE) models, the improvement remains relatively modest.

### 3.2 Results for U-Net and models from segmentation models library

For the U-Net experiments, the dataset size is limited to a maximum of 3,980 training samples and 100 test samples. This study evaluates the classic U-Net, as well as U-Net and FPN with various backbones from the Segmentation Models library. The experimental setup uses a batch size of 4 and epochs set to 50. All models are trained using the Adam optimizer (Kingma and Ba, 2017) with a learning rate of 0.001. Additionally, a decay factor of 0.5 is applied with patience set to 5, based on validation loss.

Table 3 indicates that the Mixed Loss with *L*_1_ is the most effective choice for this architecture. The U-Net demonstrates a significant improvement over the autoencoder (AE), surpassing its best-performing models, with a training duration of just over 15.8 h.

**Table 3:** Results for the experiments of U-Net written from scratch.

**Loss function**	**Training time(s)**	**MS-SSIM**	**PSNR**
*L*_1_	54,348	0.9753	31.526
*L*_2_	53,992	0.9756	31.396
*L*_*MSSSIM*_	56,248	0.9908	33.389
***L***_Mix_*L*__1__	**56,703**	**0.9939**	**37.075**
*L*_Mix_*L*__2__	56,402	0.9915	33.877

The best results are highlighted in bold.

The more advanced models from the Segmentation Models library significantly outperformed the previous models, as shown in Table 4. The best-performing model utilized EfficientNetB0 as its backbone, which also yielded the highest accuracy in evaluations beyond this study. Another advantage of this library is the reduced training time—training the best model required only approximately 4 h. Among the tested loss functions, the Mixed Loss with *L*_2_ (*L*_Mix_*L*__2__) achieved the best results; however, further experiments are necessary to determine the most effective loss function.

**Table 4:** Results for the experiments using Segmentation models library.

**Architecture**	**Loss function**	**Training time(s)**	**MS-SSIM**	**PSNR**
**U-Net(EfficientNetB0)**	***L***_Mix_*L*__2__	**14,217**	**0.9962**	**38.590**
U-Net (EfficientNetB0)	*L*_Mix_*L*__1__	37,093	0.9955	38.536
FPN (ResNet50)	*L*_Mix_*L*__2__	45,671	0.9941	35.365
FPN (EfficientNetB0)	*L*_Mix_*L*__2__	61,050	0.9950	37.956
FPN (ResNet50)	*L*_Mix_*L*__1__	44,982	0.9946	36.174
FPN (EfficientNetB0)	*L*_Mix_*L*__1__	62,267	0.9947	36.343

The best results are highlighted in bold.

### 3.3 Results for generative adversarial networks

A series of controlled experiments was conducted to determine suitable layer configurations and hyperparameters for the GAN model. For each dataset size, multiple settings were systematically evaluated, allowing us to analyze not only the best-performing models but also the impact of different loss formulations and training schedules on reconstruction quality. The key hyperparameters were selected empirically based on preliminary experiments and informed by prior work on GAN-based bone suppression, with the goal of ensuring stable convergence while preserving perceptual soft-tissue appearance and suppressing high-frequency bone edges.

All experiments used input images with a resolution of 512 × 512 × 3, with a batch size of 4 for experiments using 3,980 training samples and a batch size of 8 for smaller datasets. The evaluated loss functions were: WL = Wasserstein Loss, WL_2_ = Wasserstein Loss + *L*_2_, WL_1_PS = Wasserstein Loss + *L*_1_ + Perceptual Loss + Sobel Loss, WL_2_PS = Wasserstein Loss + *L*_2_ + Perceptual Loss + Sobel Loss, and WL_1_PS(FT) denoting the fine-tuned variant of the combined objective.

Table 5 provides a comprehensive overview of all evaluated GAN configurations across different dataset sizes, loss formulations, and training schedules. The results show a clear progression in reconstruction quality with increasing training data, highlighting the importance of dataset scale for stabilizing adversarial optimization and improving distribution-level alignment between generated and reference images.

**Table 5:** Results for the GAN experiments.

**Train/test size**	**Loss function**	**Training time (s)**	**Epochs**	**MS-SSIM**	**PSNR**
400/100	WL	206 615	2,500	0.8893	24.357
400/100	WL_2_	41 232	500	0.9876	35.619
1900/100	WL_2_P	190 805	500	0.9926	39.790
1900/100	WL_1_PS	193 345	500	0.9938	40.330
1900/100	WL_1_PS	290 017	750	0.9938	40.535
1900/100	WL_2_PS	193 435	500	0.9930	39.010
1900/100	WL_2_PS	290 505	750	0.9930	39.011
1900/100	WL_2_PS	328 840	850	0.9930	39.010
3980/100	WL_1_PS	315 105	500	0.9967	43.314
**3980/100**	**WL_1_PS**	**468 202**	**750**	**0.9968**	**44.085**
3980/100	WL_1_PS (FT)	158 590	250	0.9820	33.625
3980/100	WL_1_PS (FT)	313 590	500	0.9800	33.267

The best results are highlighted in bold.

For the smallest dataset (400 samples), the addition of the *L*_2_ term to the Wasserstein objective substantially improves both MS-SSIM and PSNR compared to the pure adversarial loss, indicating that pixel-wise supervision is essential in low-data regimes. For medium and larger datasets, extending the objective with perceptual and gradient-based constraints (WL_1_PS) consistently yields higher structural similarity and better suppression of residual bone edges compared to WL_2_ or WL_2_PS variants, which tend to produce slightly lower PSNR and MS-SSIM values despite longer training.

The best overall performance was achieved by the GAN trained on 3,980 samples using the combined Wasserstein + *L*_1_ + Perceptual + Sobel loss for 750 epochs, reaching an MS-SSIM of 0.9968 and PSNR of 44.085. Although this configuration required substantially longer training time (approximately 5.4 days), the results indicate that extended optimization with a composite loss promotes improved global structural consistency rather than merely minimizing localized pixel-wise errors.

The inferior performance of the fine-tuned variants further suggests that premature convergence or reduced training duration can negatively affect the balance between adversarial realism and structural fidelity. Overall, these findings are consistent with the observations of Rani et al. (2022), supporting the hypothesis that *L*_1_-based objectives combined with perceptual and gradient constraints provide a more suitable optimization landscape than *L*_2_-driven formulations for bone suppression tasks.

### 3.4 Comparative analysis between the models

Since the Autoencoder and U-Net models are trained at a resolution of 1, 024 × 1, 024 × 1, while the Generative Adversarial Network (GAN) models operate at 512 × 512 × 3, a direct comparison between these architectures may not provide meaningful insights. To address this, additional experiments were conducted in this section, where the best-performing AE and U-Net models were modified and retrained to match the 512 × 512 × 3 resolution.

Training the GAN model at a higher resolution of 1, 024 × 1, 024 × 3, which requires three channels, presents significant computational and time constraints. This section presents the results of these experiments and outlines the specific computational requirements for training the GAN model at this increased resolution.

A comparison of our findings with results from other studies in the field is provided at the end of this chapter. First, a direct comparison between the two models will be presented, followed by an experiment demonstrating how each model reconstructs an image from the test set, allowing for the calculation of MS-SSIM and PSNR metrics.

Table 6 indicates that the GAN model delivers the best performance, with the only notable drawback being the extended training time of approximately 5.4 days. An interesting observation is that the MS-SSIM values remain relatively high across all approaches, suggesting that image reconstruction is effectively performed. However, PSNR plays a crucial role in determining the overall visual quality of the reconstructed images.

**Table 6:** Overview of the best models.

**Model**	**Training time**	**MS-SSIM**	**PSNR**
AE + EfficientNetB0 (*L*_Mix_*L*__1__)	38,989	0.9909	33.541
U-Net (Segmentation Models + *L*_Mix_*L*__2__)	14,217	0.9962	38.590
**GAN (**WL_1_PS**)**	**468,202**	**0.9968**	**44.085**

The best results are highlighted in bold.

**Figure 8:**
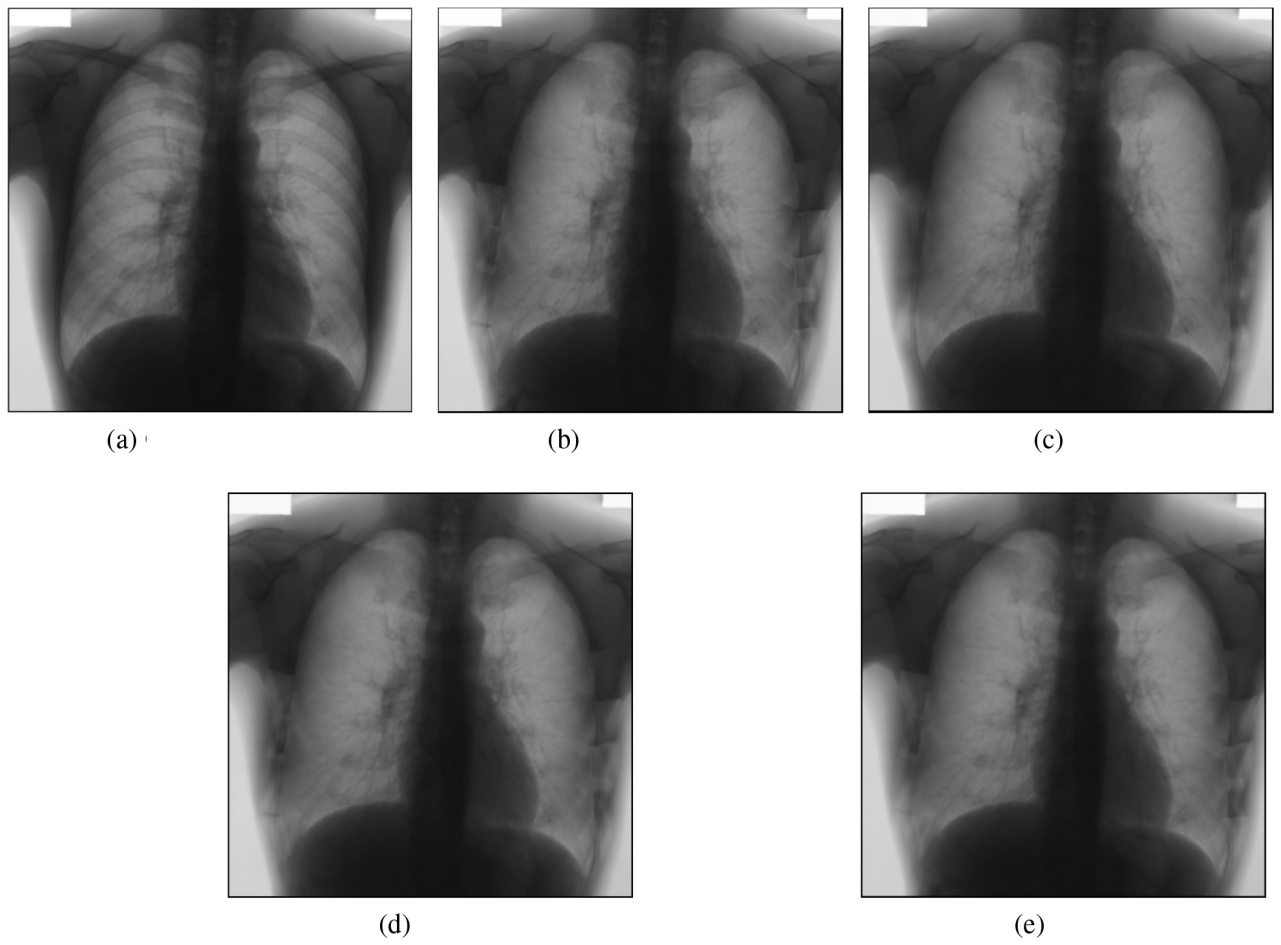
Best results for direct comparison, where: **(a)** Original image; **(b)** Target image; **(c)** AE + EfficientNetB0, MS-SSIM: 0.9821 and PSNR: 36.07; **(d)** U-Net from Segmentation models, MS-SSIM: 0.9963 and PSNR: 38.52; **(e)** GAN, MS-SSIM: 0.9985 and PSNR 45.61 (output images resized to 512 × 512).

As shown in Figure 8, visual inspection reveals that all three methods achieve effective bone suppression with comparable global image appearances. Differences between reconstructions are subtle and not clearly distinguishable without quantitative analysis. The higher PSNR and MS-SSIM values obtained by the GAN therefore reflect measurable improvements in objective reconstruction consistency rather than visually dramatic differences. To further interpret these subtle differences, a localized region-wise analysis was performed after explicit spatial alignment of all reconstructions to the reference resolution.

**Figure 9:**
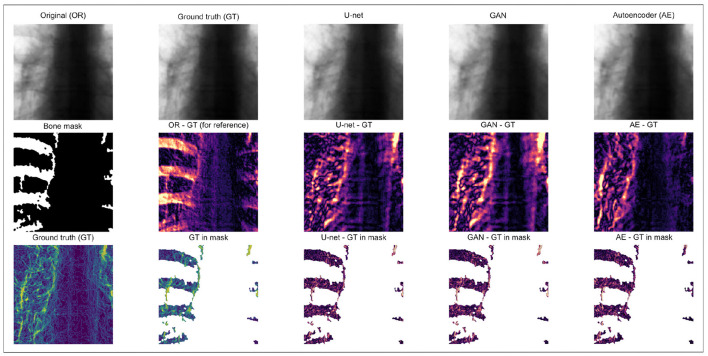
Representative case analysis of region-wise reconstruction consistency after spatial alignment. Row 1 shows the original image (OR), the reference bone-suppressed ground truth (GT), and the predictions produced by U-Net, GAN, and AE. Row 2 presents the bone mask derived from |*OR*−*GT*| together with the corresponding absolute reconstruction error maps. Row 3 visualizes the gradient magnitude of the target image and the edge error maps restricted to the bone mask. In this representative example, U-Net achieved the lowest local bone-region MAE (0.0227), while GAN exhibited a slightly higher local deviation (0.0264). However, the GAN demonstrated comparable edge-consistency behavior within masked regions and a more homogeneous distribution of residuals across the image. Despite the slightly higher local errors, GAN achieved the best global PSNR and MS-SSIM values, indicating improved overall structural consistency rather than strict minimization of localized pixel-wise deviations.

To further interpret the subtle differences observed in Figure 8, a representative case analysis was conducted after the explicit spatial alignment of all predictions to the reference resolution (Figure 9). A bone mask derived from the absolute difference between the original and reference bone-suppressed image (|*OR*−*GT*|) was used to separate bone and non-bone regions, and reconstruction errors were evaluated relative to the target image.

Within the masked bone region, U-Net achieved the lowest local mean absolute error (MAE_*bone*_ = 0.0199), followed by AE (0.0206), while GAN exhibited slightly higher local deviation (0.0222). Outside the bone mask, residuals produced by the GAN were more uniformly distributed, indicating balanced reconstruction across the entire image. Edge-based analysis revealed only minor differences among the models within masked regions.

These findings highlight a fundamental optimization distinction. U-Net, trained predominantly with *L*_1_/*L*_2_-driven objectives, favors pixel-wise intensity agreement, leading to slightly lower localized absolute error. In contrast, the GAN integrates adversarial and perceptual components that promote structural coherence and distribution-level consistency. Consequently, although U-Net may minimize local absolute deviation in specific regions, the GAN attains superior global PSNR and MS-SSIM values, reflecting improved overall reconstruction balance rather than isolated pixel-level similarity.

Within the masked bone regions, the U-Net achieved a slightly lower local absolute reconstruction error, indicating closer pixel-wise intensity matching to the reference. In contrast, the GAN did not consistently minimize local absolute deviation within these regions; however, it exhibited a more homogeneous distribution of residuals across both bone and non-bone areas. This behavior suggests that the GAN does not primarily optimize strict pixel-level similarity but rather promotes globally balanced structural reconstruction.

From an optimization perspective, this behavior reflects the fundamental distinction between pixel-wise reconstruction objectives and adversarial-perceptual formulations. The U-Net, trained predominantly with pixel-wise reconstruction losses (e.g., *L*_1_/*L*_2_ loss), promotes strict intensity agreement at the local level, leading to slightly lower localized absolute error. In contrast, the GAN integrates adversarial and perceptual components that encourage structural coherence and distribution-level consistency across the entire image. Consequently, although U-Net may minimize local absolute deviation in specific regions, the GAN attains superior global PSNR and MS-SSIM values, reflecting improved overall reconstruction balance rather than isolated pixel-level similarity.

### 3.5 Comparative analysis for 512 × 512 input size

To enable a direct comparison, the performance of models trained at the same resolution of 512 × 512 will be evaluated. Following this, an experiment will be conducted to analyze the reconstructed images, highlighting differences in MS-SSIM and PSNR on a test sample.

The training parameters for the autoencoder and U-Net models were set to a batch size of 4 and 50 epochs.

Table 7 shows that there is no significant difference in performance between models trained at 1, 024 × 1, 024 and those trained at 512 × 512. Once again, the GAN model achieved the best results, with consistently high MS-SSIM values across all cases. The most notable differences appear in PSNR, which can be visually examined alongside the original and target images.

**Table 7:** Direct comparison of the best models trained on images of size 512 × 512.

**Model**	**Training time**	**MS-SSIM**	**PSNR**
AE (*L*_Mix_*L*__1__)	4,803	0.9837	31.703
U-Net (*L*_Mix_*L*__1__)	14,067	0.9939	37.069
U-Net (segmentation models + *L*_Mix_*L*__2__)	9,706	0.9955	37.592
**GAN (**WL_1_PS**)**	**468,202**	**0.9968**	**44.085**

The best results are highlighted in bold.

The results for the 512 × 512 models are summarized in Table 8. The reported metrics correspond to a single representative test image that was not included in the augmented JSRT dataset and are provided to illustrate the qualitative behavior of the best-performing configurations under identical spatial resolution. Across all evaluated paradigms, the GAN-based model achieved the highest MS-SSIM and PSNR values. Additionally, the FPN-based model exhibited unstable training behavior under the given experimental configuration, leading to the emergence of noticeable image artifacts in the reconstructed outputs. Since the FPN architecture was not part of the three primary modeling paradigms evaluated in this study (autoencoder, U-Net, and GAN), it was not analyzed further and is therefore not considered in the main comparative analysis.

**Table 8:** Direct comparison of the best-performing models trained on images of size 512 × 512, evaluated on a single representative test image not included in the augmented JSRT dataset.

**Model**	**MS-SSIM**	**PSNR**
c) AE (*L*_Mix_*L*__1__)	0.9804	33.602
d) U-Net (*L*_Mix_*L*__1__)	0.9919	37.247
e) U-Net (segmentation models + *L*_Mix_*L*__2__)	0.9957	40.014
**f) GAN (**WL_1_PS**)**	**0.9985**	**45.611**

The best results are highlighted in bold.

## 4 Discussion

In this study, three methodologies previously applied to this task were implemented and rigorously evaluated: autoencoders (AE), U-Net, and generative adversarial networks (GAN). Each of these methods operates on a distinct principle, making this study not only an assessment of their effectiveness but also a comparison to determine the most suitable approach for this task. Autoencoders and U-Net, as non-generative models, tend to be less susceptible to spurious noise introduced during training or inference. However, the quality of the reconstructed images may sometimes be suboptimal, potentially complicating the diagnosis based on the X-ray output. In contrast, generative adversarial networks, due to their inherently generative nature, can exhibit greater sensitivity to such noise; yet, they outperformed the other approaches overall based on quantitative metrics. Each architecture was systematically designed and evaluated across a range of parameters, with the optimal configurations selected for the final assessment. It is essential to emphasize that chest radiographs require interpretation by a qualified healthcare professional for accurate clinical diagnosis.

One of the first methods to address the bone (rib) suppression challenge was the multiresolution massive training artificial neural network (MTANN) (Suzuki et al., 2006), which employed decomposition/composition techniques and was trained on chest radiographs and dual energy bone images. This method produced “bone-image-like” outputs, enabling the creation of “soft-tissue-image-like” images with effectively suppressed ribs and clavicles. Despite such advancements, bone removal in radiological imaging remains a significant challenge, as evidenced by numerous recent studies focusing on refining existing methods, comparing them, optimizing workflows, and addressing limitations to improve usability for radiologists and physicians. A major challenge in the bone suppression task is the scarcity of bone suppressed data and the general lack of publicly available datasets, as reported (Ren et al., 2021; Schiller et al., 2025). The state-of-the-art methods include deep learning architectures based mainly on autoencoders (AE), U-Net, and, more recently, generative adversarial networks.

Convolutional denoising autoencoders were chosen for this task because ribs can be treated as structured interference rather than random noise. This learning paradigm enables the network to map input radiographs containing bone structures to bone-suppressed target images by minimizing a reconstruction loss function. This unsupervised machine learning technique was used because it learns to map corrupted data (bones) to uncorrupted data (bone-suppressed images) by minimizing a loss function. This approach has previously been used by Gusarev et al. (2017) and Kalisz and Marczyk (2021). Our results indicate that the best performance is achieved by the autoencoder with a pre-trained encoder (FPN + EfficientNetB0) (Table 2). However, the performance improvement is not as significant as with the autoencoder implemented from scratch (Table 1). In direct comparison to other methods, the autoencoder achieved the worst results (Table 8). Compared to the implementation by Gusarev et al. (2017), our AE-based approach with a pre-trained encoder achieved a better MS-SSIM value (Table 9).

**Table 9:** Comparison of the models with other articles.

**Name**	**Model and technique**	**MS-SSIM**	**PSNR**
Gusarev et al. (2017)	Autoencoder and convolutional layers	0.907	-
Eslami et al. (2020)	Conditional GAN and dilated convolutions	0.970	-
Chen et al. (2019)	Cascade of multiscale CNN	0.977	39.40
Zhou et al. (2020)	Multi-scale and conditional adversarial network	0.884	39.7
Huang et al. (2020)	U-Net3+	0.985	33.69
Kalisz and Marczyk (2021)	Autoencoder and convolutional layers	0.982	32.60
Rajaraman et al. (2021)	Residual network model (ResNet-BS)	0.949	34.07
Ziviani et al. (2022)	Conditional GAN	0.943	34.97
Rani et al. (2022)	SFRM - GAN	0.989	42.83
Zunair and Hamza (2021)	U-NetSharp	0.985	33.72
Schiller et al. (2025)	xU-NetFullSharp	0.986	33.81
*Our approach*	*AE + EfficientNetB0 (*L*_Mix_*L*__1__)*	*0.991*	*33.54*
*Our approach*	*U-Net (Segmentation Models + *L*_Mix_*L*__2__)*	*0.996*	*38.59*
**Our approach**	**WL_1_PS**)	**0.997**	**44.09**

The best results are highlighted in bold.

The U-Net (Ronneberger et al., 2015) is a widely used neural network in medical imaging, mainly designed for image segmentation (Siddique et al., 2021). Due to its adaptability, various modifications and integrations have been applied to bone suppression workflows in chest radiographs (Li et al., 2020; Xu et al., 2024; Schiller et al., 2025), making it a relevant choice for this study.

Schiller et al. (2025) developed xU-NetFullSharp, an advanced deep learning model for bone suppression based on the U-NetSharp architecture, originally designed for medical image segmentation (Zunair and Hamza, 2021). The xU-NetFullSharp improves performance by incorporating multi-scale skip connections from shallow layer blocks to deeper layers, enhancing the model's ability to retain low-level contextual information. They also implemented several U-Net-based architectures using TensorFlow and Keras, in combination with NumPy and OpenCV, including U-Net, Attention U-Net, U-Net++, Attention U-Net++, U-Net3+, U-NetSharp, and xU-NetFullSharp. Subsequently, these architectures were compared with the latest models for bone suppression, namely the autoencoder proposed by Kalisz and Marczyk (2021) and the ensemble model DeBoNet (Rajaraman et al., 2022).

Our U-Net-based approach, which integrates Segmentation Models with a Mixed *L*_2_ Loss function, outperformed xU-NetFullSharp in the reported evaluation metrics. Specifically, our model achieved an MS-SSIM of 0.996 and a PSNR of 38.59, while xU-NetFullSharp obtained an MS-SSIM of 0.986 and a PSNR of 33.81 (Table 9). Compared directly to the autoencoder, our U-Net-based architecture demonstrated superior MS-SSIM and PSNR values but was outperformed by the GAN, which achieved the highest results in both metrics (Table 8).

Oh and Yun (2018) used a denoising approach based on conditional image-to-image translation with adversarial training within a GAN framework to model the conditional probability distribution of the output (bone-suppressed chest radiograph) given the input (original chest radiograph). In addition, Liang et al. (2020) demonstrated that GANs could effectively learn the nuances of bone suppression using adversarial learning techniques, resulting in images that preserve essential soft tissue details while minimizing bone artifacts. To improve image quality, GANs facilitate the generation of bone-suppressed images in a more automated and efficient manner. Techniques such as CycleGAN and conditional GAN have been employed to enhance the bone suppression process, allowing the simultaneous generation of bone-free images and organ segmentation (Zhou et al., 2020; Eslami et al., 2020).

In 2020, Zhou et al. (2020) incorporated enforced semantic features into a dilated conditional GAN, which allows for a better contextual understanding of the images being processed. This integration not only improves the quality of the generated images but also aligns the output more closely with clinical expectations. Their model was trained by enforcing the pixel-wise intensity similarity and the semantic-level visual similarity between the generated radiograph and the ground truth by optimizing an *L*_1_ loss of the pixel intensity values on the generator side and a feature matching loss on the discriminator side. Since a naïve combination of adversarial loss and pixel-wise *L*_1_ loss failed to capture the semantic information of radiographs, a feature matching loss was introduced on top of the discriminator. This approach allowed the discriminator to extract feature representations from each pair of real and generated images using activations from its fourth convolutional block and to compute the mean of the element-wise *L*_1_ distance between them. the resulting loss function for optimizing the discriminator was defined as $D^{*}=L_{adv_{D}}+\lambda_{fm}L_{fm}$, where *L*_*ad*_*v*__*D*__ is the adversarial loss of the discriminator, *L*_*fm*_ is the feature matching loss, and λ_*fm*_ is a constant to adjust the weight of the feature matching loss. The utilization of feature matching loss enforced the similarity of high-level feature maps.

Our GAN architecture, enhanced by the incorporation of Wasserstein Loss along with *L*_1_ loss, Perceptual Loss, and Sobel Loss functions, delivered the best performance. Its metrics, including a PSNR of 44.09 dB and an MS-SSIM of 0.9968, confirm the model's ability to suppress bone structures effectively while preserving high image quality and critical soft tissue details. Compared to other studies (Ziviani et al., 2022; Rani et al., 2022; Eslami et al., 2020) evaluated on the same dataset, our method achieved the highest reported MS-SSIM (0.997) and PSNR (44.09) values under the reported experimental settings.

Despite achieving the best quantitative performance in our experiments, the GAN-based model is associated with a higher computational cost compared to the autoencoder and U-Net architectures. This reflects the inherent complexity of adversarial learning and the composite loss formulation, representing a methodological trade-off rather than an implementation limitation. In practical deployments, training time could be reduced through multi-GPU parallelization, mixed-precision training, or resolution-aware strategies, facilitating scalable application in larger datasets or clinical workflows.

This study is limited to evaluation on the publicly available JSRT dataset and its augmented variants. Although augmentation increases training variability, it does not introduce patient-level diversity or independent acquisition conditions. The findings should therefore be interpreted as methodological benchmark results rather than evidence of clinical generalizability, and validation on independent multi-center datasets and external test sets remains necessary to assess robustness under heterogeneous real-world acquisition conditions.

## 5 Conclusion

The increased visibility of the lung regions in bone-suppressed images can aid in the inspection of soft-tissue structures and may support downstream computer-aided analysis, while dedicated clinical validation is required to assess the diagnostic impact. This suggests that the proposed GAN configuration may represent a promising methodological direction for medical imaging preprocessing.

The presented GAN optimization integrates a combined objective composed of Wasserstein loss together with pixel-wise, perceptual, and gradient-based components (*L*_1_, Perceptual loss, and Sobel loss), aiming to balance intensity fidelity, structural consistency, edge preservation, and distribution-level realism in the generated outputs. This study was designed as a methodological evaluation of modeling strategies rather than a clinical validation study.

In addition to the GAN-based approach, we also trained models for bone removal using autoencoders and a custom implementation of the U-Net architecture from scratch. However, their performance across the evaluated metrics was consistently lower compared to the proposed GAN model, highlighting the advantage of integrating multiple loss components in this context.

The source code and trained models developed in this work are publicly available to the research community on GitHub, promoting transparency, reproducibility, and further development in this important area.

Future studies could explore its integration into clinical workflows, where collaboration with radiologists can provide critical insights into its practical impact on diagnosis. In addition, future advances, including optimized training strategies, refined loss functions, and adaptation to other imaging modalities that demand high detail denoising and feature preservation, could further enhance the utility of this approach. The findings of this research provide a methodological reference point for the further development of bone suppression models within computer-aided imaging workflows.

## Data Availability

Publicly available datasets were analyzed in this study. The data can be found at: http://db.jsrt.or.jp/eng.php. The data and code supporting the findings of this study are openly available as a reproducible package at Zenodo: Jochymek, L., Vašinková, M., and Gajdoš, P. (2026). GAN-based bone suppression using a combined loss function (v1.0). doi: 10.5281/zenodo.18845760.
